# Research Progress in the Production of D-Lactic Acid from Renewable Resources

**DOI:** 10.3390/foods15152753

**Published:** 2026-08-05

**Authors:** Bingyi Tao, Qi Lin, Ren He, Tingting Huang, Shaoxiong Liang, Hongkun Chen, Xiaoping Rao, Xuchong Tang, Jianchun Jiang

**Affiliations:** 1 College of Chemical Engineering, Huaqiao University, Xiamen 361021, China; 2 Academy of Advanced Carbon Conversion Technology, Fujian Provincial Key Laboratory of Biomass Low-Carbon Conversion, Huaqiao University, Xiamen 361021, China; 3 BaYeCao Health Industry Research Institute Co., Ltd., Xiamen 361021, China; 4 Institute of Chemical Industry of Forest Products, Chinese Academy of Forestry, Nanjing 210042, China

**Keywords:** D-lactic acid, renewable sources, microbial fermentation

## Abstract

D-lactic acid is a key chiral intermediate widely applied in agriculture, pharmaceuticals, and polylactic acid synthesis, with microbial fermentation as its main production method. Commercial D-lactic acid production depends heavily on refined carbohydrates and yeast extract as carbon and nitrogen sources, resulting in prohibitive raw material costs. Abundant in microbially available proteins and carbohydrates, renewable resources can act as carbon and nitrogen substrates for efficient D-lactic acid production. Repurposing these resources reduces production expenses and is essential for the industrial scaling of D-lactic acid manufacturing. This paper reviews research advances in D-lactic acid fermentation using renewable feedstocks including agricultural wastes, sugar industry residues, oil processing wastes, and cheese processing by-products as carbon and nitrogen sources.

## 1. Introduction

Facing environmental problems and depleting fossil fuels amid growing global energy demand, there is an urgent need for today’s industrial sector to shift from reliance on fossil fuels to renewable energy sources. As a representative bio-based polymer, polylactic acid (PLA) is widely recognized as a highly promising sustainable material owing to its inherent biodegradability, favorable physicochemical properties, and competitive projected production costs [1]. PLA is exclusively polymerized from lactic acid monomers, which exist as two enantiomers: L-lactic acid and D-lactic acid. L-lactic acid and D-lactic acid have identical physical and chemical properties in environment but differ markedly in polymer performance and industrial application positioning. Stereocomplex PLA formed by blending L-PLA and D-PLA exhibits a melting temperature of 220–230 °C, roughly 50 °C higher than neat PLA homopolymer, with markedly improved mechanical strength and heat resistance [2]. Therefore, D-lactic acid acts as an irreplaceable chiral building block for the fabrication of stereocomplex PLA due to the presence of L-lactate dehydrogenase (L-LDH) in the human body, which can metabolize L-lactic acid, which is often used in the food industry. D-lactic acid, due to its unique chiral structure, is widely applied in agriculture [3], medicine [4], and the chemical industry [5], including for the production of PLA [6]. However, the industrial manufacturing of D-lactic acid remains far less technologically mature than that of L-lactic acid because high-titer, high-optical-purity native D-lactic acid strains are scarce, and D-LDH exhibits lower catalytic efficiency [7]. D-lactic acid processes remain at laboratory or pilot scale, restricting further industrial scaling.

At the industrial scale, lactic acid is mainly produced through chemical synthesis processes and microbial fermentation. However, the chemical synthesis pathway unavoidably forms a racemic mixture of stereoisomers, whereas enantiomerically pure L-lactic acid or D-lactic acid can be synthesized by microbial fermentation. The use of chemical methods to prepare lactic acid involves highly toxic raw materials, potential safety hazards in the production process, and the resulting product being a racemic mixture. However, microbial fermentation has low environmental pollution, mild production conditions, fewer by-products, and can utilize organic waste and renewable resources. Therefore, in industrial D-lactic acid production, microbial fermentation is mostly used [8]. Microbial fermentation can be used to produce high-optical-purity D-lactic acid by fermentation using certain bacteria with high-optical-purity D-lactic acid production capacity, such as *Bacillus* spp., *Lactobacillus* spp., *Streptomyces* spp., etc. [9]. Common D-lactic acid producing bacteria include *Lactobacillus delbrueckii*, *Lactobacillus plantarum*, *Sporolactobacillus inulinus*, etc. [10]. They can be categorized as wild, mutagenic, genetically engineered, and adaptive evolution engineered based on whether or not the fermentation strain is modified. Among them, genetically engineered and evolutionary engineered bacteria are the more widely used strains. Fermentation systems for D-lactic acid can be generally classified into four distinct operational patterns, covering batch fermentation, fed-batch fermentation, continuous fermentation, and cell-recycle fermentation. The advantages and disadvantages of the four modes of fermentation are compared in Table 1.

Industrial-scale manufacturing processes for L-lactic acid have attained technological maturity, whereas D-lactic acid biosynthesis is still confined to laboratory stage. The high cost of substrate represents the core limiting factor for D-lactic acid industrialization [15]. Currently, commercial lactic acid production relies predominantly on refined carbohydrate feedstocks as core carbon substrates, including glucose, starch, and sucrose extracted from sugarcane and sugar beet roots, directly contributing higher manufacturing costs [1]. This has prompted the lactic acid production industry to look for cheap and renewable alternative substrates. In fact, most agro-industrial wastes and their by-products contain nutrients such as proteins, lipids, carbohydrates [16], such as lignocellulose, algae, whey, molasses, corn stover, and household wastes renewable resources [1]. The main renewable resources of the agro-industry include agricultural waste, by-products of the sugar industry, the cheese-processing industry, biodiesel production, the oil-processing industry, and so on. Agricultural waste includes corn stovers, corn cobs, etc., sugar industry by-products include molasses, bagasse, etc., and cheese processing industry by-products include cheese whey powder, whey permeate, etc. Oil industry by-products include soybean meal, peanut meal, cottonseed, etc. Microorganisms can utilize these renewable resources to produce high-optical-purity D-lactic acid through fermentation [17]. However, most existing reviews have focused on strategies for high-titer D-lactic acid production, typically limited to one or a small number of sustainable feedstocks as a means of lowering fermentation costs. Comprehensive reviews that systematically evaluate D-lactic acid conversion technologies across diverse renewable resources, contextualized within the latest advances in synthetic biology, remain scarce. This work presents the first thorough, systematic overview of progress in D-lactic acid biosynthesis from a wide range of renewable feedstocks. We further outline future research prospects, with the goal of providing a holistic reference and fresh research perspectives for investigators in this field.

## 2. Production of D-Lactic Acid from Renewable Resources

**Figure 1:**
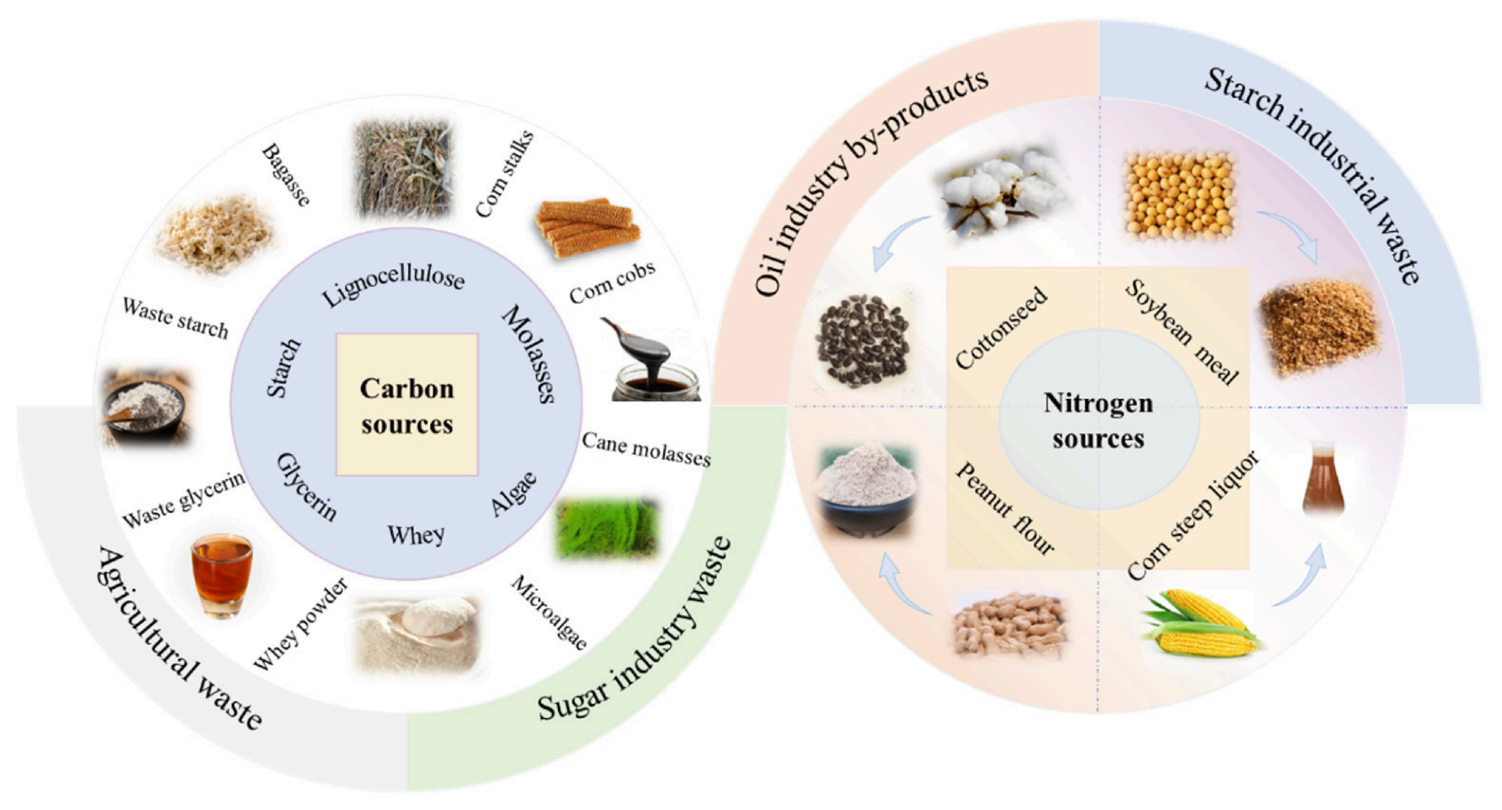
Renewable resources as carbon and nitrogen sources for the production of D-lactic acid.

In conventional lactic acid fermentation processes, substrate costs typically account for 40 to 70 percent of overall production expenditure. Reducing raw material input costs is therefore essential to improving the economic efficiency of lactic acid production. Studies have demonstrated the feasibility of using agricultural wastes such as lignocellulose [18], molasses [19], whey [20], and algae [21] as carbon sources for D-lactic acid fermentation, and oil industry wastes such as cottonseed, soybean meal, corn steep liquor and peanut meal as nitrogen sources for fermentation. Figure 1 shows the renewable resources available for D-lactic acid production, and their specific applications will be introduced in the following text.

### 2.1. Production of D-Lactic Acid Using Renewable Resources as a Carbon Source

In industrial D-lactic acid fermentation, the high cost of refined glucose leads to increased overall fermentation costs when it is used as the primary carbon source. Therefore, cheaper renewable raw materials or wastes are needed to replace glucose. Alternative substrates include disaccharides like sucrose and maltose, and polysaccharides like starch, cellulose and hemicellulose [22]. Renewable resources rich in these sugars include agricultural wastes such as corn cobs, corn stover, bagasse, and wheat bran, sugar mill wastes such as molasses and beet pulp [10], and dairy industry by-products such as cheese whey [23]. This section focuses on the research progress on the use of renewable resources such as lignocellulose, molasses, starch, whey, and algae as carbon sources for D-lactic acid fermentation (Table 2).

**Figure 2:**
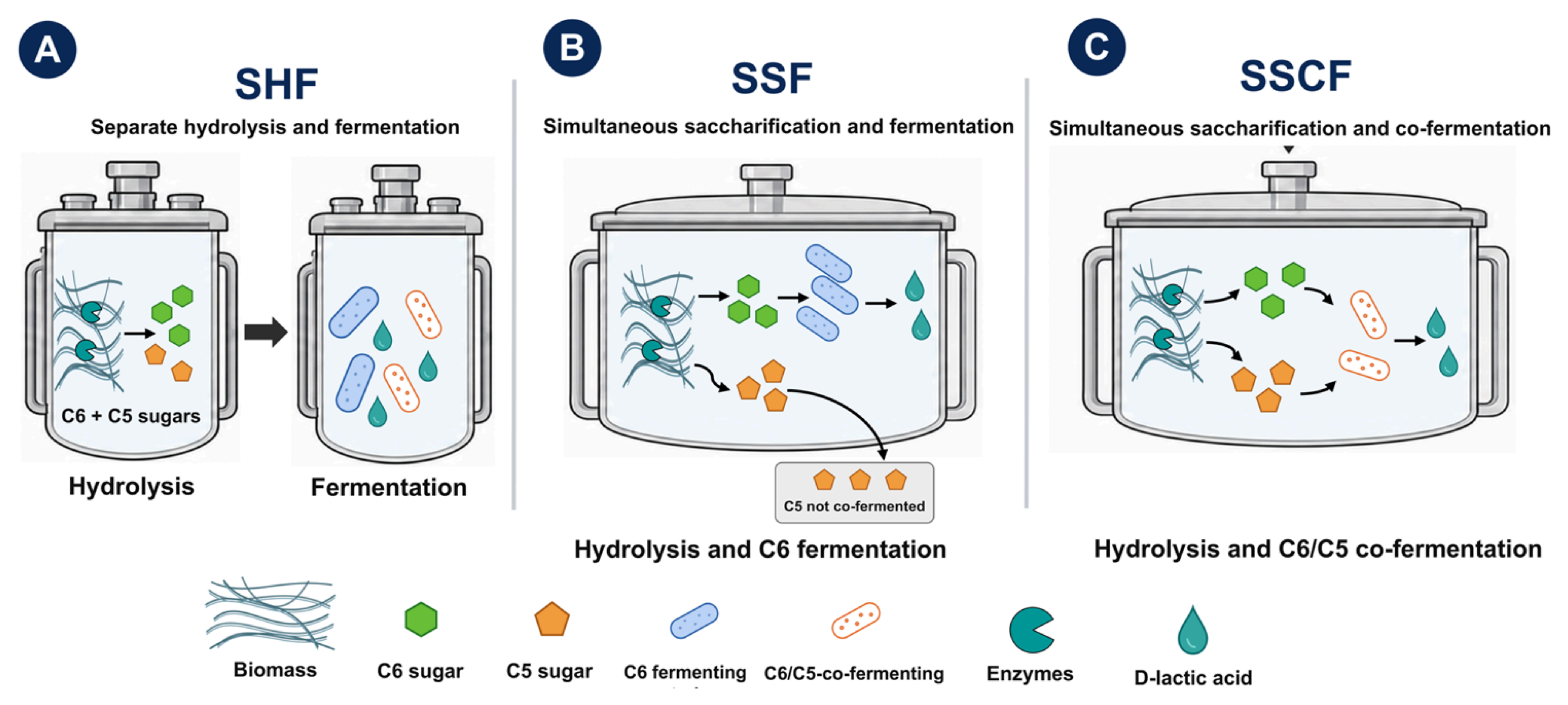
(**A**) Separate hydrolysis and fermentation (SHF), (**B**) simultaneous saccharification and fermentation (SSF), and (**C**) simultaneous saccharification and co-fermentation (SSCF).

Usually, renewable resources need to be chemically hydrolyzed or enzymatically hydrolyzed into fermentable sugars before they can be utilized by strains. The fermentation process can be classified into separate hydrolysis and fermentation (SHF), simultaneous saccharification and fermentation (SSF), and simultaneous saccharification and co-fermentation (SSCF) based on whether the hydrolysis and fermentation processes proceed synchronously and whether multiple sugars are utilized simultaneously (Figure 2) [24]. SHF involves first hydrolyzing polysaccharides such as cellulose into fermentable monosaccharides such as glucose, and then converting the monosaccharides into lactic acid. For the SHF, its hydrolytic and fermentative phases can be performed separately under the optimal temperature and pH conditions, but the sugar released during the hydrolysis process will inhibit enzyme activity and there are drawbacks such as a long fermentation period, and easy contamination of bacterial strains [25]. For the SSF and SSCF, hydrolytic degradation and fermentative conversion occur simultaneously in a single reactor, so that strains can directly utilize the end product of hydrolysis (simple sugars or monosaccharides). In contrast, SSF and SSCF uses simple equipment, has a short fermentation cycle, is highly productive, and effectively reduces product inhibition, but there are usually differences in the optimal temperature and pH for hydrolysis and fermentation [26].

**Table 2:** Research progress of renewable resources for carbon source in D-lactic acid fermentation.

Substrate	Strains	Fermentation Mode	Titer (g/L)	Productivity (g/L/h)	Optical Purity (%)	Reference
Wheat straw	Adaptively evolved *P. acidilactici* ZY271	SSCF (250 mL)	128	-	99.1	[27]
Sugarcane bagasse	*P. acidilactici* XH11	SSCF	57.0	0.79	-	[28]
Corn stover	*P. acidilactici* PA204	SSF	104.11	1.24	-	[29]
Corn stover	Adaptively evolved *P. acidilactici* ZY15	SSCF (250 mL)	97.3	1.01	99.2	[30]
Corn stover	*L. plantarum* NCIMB 8826	Fed-batch (2 L)	61.4	0.32	99	[31]
Corn stover	*P. acidilactici* ZP26	SSF (5 L)	76.76	-	99.32	[32]
Corn stover	*L. delbrueckii* subsp. *bulgaricus* ATCC11842	SSF	18	-	99	[33]
Corn stover	*S. inulinus* YBS1-5	-	70.7	0.65	-	[18]
Corncob slurry	*P. acidilactici* XH11	SSCF (500 mL)	61.9	0.64	-	[34]
Molasses	*L. delbrueckii*	Fed-batch (750 mL)	162	3.37	97.66	[35]
Sugarcane molasses	*L. delbrueckii*	Fed-batch (7.5 L)	112.3	2.4	99.6	[36]
Molasses/Corncob residue hydrolysate	*Z. mobilis*	Batch (50 mL)	42.8	0.6	-	[37]
Sugarcane molasses	*K. marxianus*	Batch (1 L)	66.26	0.92	-	[38]
Corn flour hydrolyzate	*S. inulinus* Y2-8	Fed-batch (7.5 L)	218.8	1.62	99.0	[39]
Corncob residue	*S. inulinus* YBS1-5	Fed-batch (7 L)	107.2	1.19	99.2	[40]
Rice straw	*L. delbrueckii*	Membrane integrated continuous fermentation	-	18.56	99.5	[14]
Pulp mill residue	*L. coryniformis* subsp. *torquens*	SHF (1.1 L)	56	2.8	-	[41]
Delignified hardwood pulp	*L. plantarum* mutant	SSF (100 mL)	102.3	2.61	99.2	[42]
Dried distiller’s grains with solubles	*L. coryniformis* subsp. *torquens*	SSF (1.5 L)	24.1	-	99.9	[25]
Sugar beet pulp hydrolysate	*L. coryniformis* subsp. *torquens* DSM 20005	Fed-batch (2 L)	40	0.86	99.4	[43]
Orange peel wastes	*L. delbrueckii* ssp. *delbrueckii*	Batch (100 mL)	-	2.35	-	[44]
Kodo millet bran residue	*L. delbrueckii* ssp. *delbrueckii* NBRC 3202	Batch (250 mL)	25.38	0.26	97.79	[45]
Palmyra palm jaggery	*S. inulinus* NBRC 13595	Batch	189	5.25	99.6	[46]
Waste *Curcuma longa* biomass	*L. paracasei*	SSCF (500 mL)	91.61	2.08	-	[47]
Raw corn starch	*L. plantarum*	2 L	73.2	-	99.6	[48]
Brown rice	Engineered *L. plantarum* NCIMB 8826	SSF (3 L)	117.1	0.81	99.6	[49]
Cheese whey powder	*L. bulgaricus* CGMCC 1.6970	Fed-batch (3 L)	113.18	2.36	-	[50]
Whey permeate	*L. delbrueckii/ L. lactis*	Batch (2.4 L)	45	-	-	[51]
Dry biomass of *H*. *reticulatum*	*L. coryniformis* subsp. *torquens*	SSCF (5 L)	36.6	2.38	95.8–99.6	[52]
Dry biomass of *G. amansii*	*P. acidilactici*	SSCF	41.4	0.29	-	[21]
Lyophilised biomass of *A. platensis* F&M-C256	*L. plantarum ATCC 8014*	Batch (50 mL)	3.7	0.05	-	[53]
Crude glycerol	*E. coli*	Batch	115	3.29	99	[54]
Inulin	*L. bulgaricus* CGMCC 1.6970	SSF (250 mL)	123.6	1.72	99.9	[55]
Jerusalem artichoke tuber powder	*K. marxianus* BY25571	Batch (5 L)	122	-	99	[56]

“-”: Indicates that the relevant content was not found.

#### 2.1.1. Lignocellulose

Lignocellulose is mainly derived from forestry wastes such as wood and branches, agricultural wastes such as straw and corn cobs, and cellulose wastes from manufacturing and agriculture. It is an excellent alternative to traditional fermentation carbon sources due to its low cost, rich source, and polysaccharide content [26]. Lignocellulose features a rigid composite architecture with strong recalcitrance to degradation, and this inherent structural property impedes the efficient enzymatic hydrolysis of cellulose and hemicellulose components. Therefore, the conversion of lignocellulosic biomass into D-lactic acid involves a biorefinery process comprising pretreatment, detoxification, hydrolysis, fermentation, and product recovery [26]. Widely adopted pretreatment technologies for lignocellulosic biomass are generally grouped into four primary categories including physical, chemical, physicochemical and biological methods, with the combination of physical, chemical, and enzymatic hydrolysis being the most widely used. Detoxification eliminates pretreatment-derived inhibitors such as furfural and phenolic compounds to avoid cell growth inhibition, with common approaches including filtration and biodetoxification [57]. Hydrolysis breaks down cellulose and hemicellulose into fermentable monosaccharides, and enzymatic hydrolysis is the predominant strategy for its high sugar yield and mild reaction conditions [58]. Fermentation is the core bioconversion step, where strains metabolize released sugars into the target product under controlled pH and temperature [59]. The final product recovery stage covers downstream purification processes that directly determine the final chemical and optical purity of D-lactic acid, as well as the overall process economics [60].

However, pretreated lignocellulosic hydrolysate contains fermentation inhibitors such as furfural, 5-hydroxymethylfurfural, furan derivatives, organic acids, and other by-products which will affect microbial growth and fermentation [61]. To mitigate the inhibitory effects of these compounds, adaptive laboratory evolution represents an effective approach to enhance the inhibitor tolerance of production strains. Qiu et al. [34] performed 111 days of adaptive laboratory evolution of an initial strain of *P. acidilactici* XH11 in undetoxified corncob prehydrolysates, and after three phases of adaptive evolution, the adapted strain produced D-lactic acid in a titer that was 126.8% higher than that of the parental strain. In one-pot SSCF using the undetoxified, pretreated whole slurry of sugarcane bagasse, 57.0 g/L D-lactic acid was produced by *P. acidilactici* XH11 [28]. While adaptive laboratory evolution substantially enhances the inhibitor tolerance of production strains, the entire strain evolution process generally requires a lengthy experimental cycle. Therefore, in addition to adaptive laboratory evolution of strains, suitable detoxification methods can be selected to remove inhibitors from lignocellulosic hydrolysates to improve the efficiency of subsequent hydrolysis and fermentation [62]. Commonly used detoxification methods include ion exchange absorption, water washing, vaporization, etc. Currently, there is a biological detoxification method using *Amorphotheca resinae* ZN1, which has excellent detoxification performance without generating wastewater. He et al. [27] used a highly selective biodetoxifying fungus, *A. resinae* ZN1, to remove inhibitor impurities from dry-acid-pretreated wheat straw fermentation broth and effectively improve D-lactic acid titer. Qiu et al. [28] found that a pre-fermentation step before H_2_SO_4_ pretreatment converted 98% of water-soluble carbohydrates to D-lactic acid, greatly reducing furfural formation and boosting D-lactic acid titer.

In addition, the low conversion rate of lignocellulose to D-lactic acid is often a challenge for its use. Thus, promoting efficient xylose consumption in corresponding hydrolysates is a core measure to achieve higher overall substrate conversion performance. The glucose content in lignocellulosic hydrolysates is 60–70%, and the xylose content is 30–40%. Xylose is a pentose that is widely present in hemicellulose in the form of xylan. When glucose and other fermentable sugars are present simultaneously, lactic acid bacteria (LAB) will preferentially convert glucose, and will utilize other monosaccharides after glucose is exhausted. This phenomenon is called carbon catabolite repression (CCR) [63]. The CCR resulted in low xylose consumption rates and thus low D-lactic acid concentration and productivity, and most LAB were unable to efficiently convert xylose from lignocellulose. To address this problem, researchers have improved xylose utilization of strains through genetic engineering and evolutionary engineering techniques. For example, Lu et al. [64] genetically engineered *Escherichia coli* with hexose and pentose assimilative capacity and used adaptive laboratory evolution obtain the evolved strain *E. coli* JH15, which efficiently eliminated CRR and increased the xylose consumption rate and the D-lactic acid production rate. Qiu et al. [30] reported that a xylose co-fermenting *P. acidilactici* ZY15 was developed by engineering a xylose assimilation pathway (via chromosomal integration of screened *xylA* and *xylB*) and adaptive evolution. It achieved 97.3 g/L D-lactic acid and 92.6% xylose conversion in high solid SSCF of pretreated and biodetoxified corn stover. He et al. [27] reported that the *P. acidilactici* ZY271 strain obtained through adaptive laboratory evolution realized synchronous co-fermentation with balanced consumption rates for glucose, xylose, arabinose, mannose and galactose derived from wheat straw under the SSCF operation regime, and the accumulated D-lactic acid reached 128 g/L with an optical purity of 99.1%. Meanwhile, fermentation can be carried out using strains that efficiently utilize xylose. Zhang et al. [29] produced D-lactic acid by SSF using *P. acidilactici* PA204, which is resistant to high temperatures and efficiently utilizes xylose, thereby enabling effective conversion of NaOH-pretreated corn stover into D-lactic acid.

The selection of appropriate pretreatment, detoxification methods and the use of genetic engineering techniques to modify strains can effectively improve lignocellulosic D-lactic acid conversion. But the application of lignocellulose to industrial-scale D-lactic acid production is difficult, including high pretreatment costs, high enzyme costs, insufficient microbial tolerance to degradation inhibitors, low efficient co-utilization of mixed sugars, uneven mass transfer from high solid content, crude raw material contamination, and reactor damage during pretreatment [28,65,66,67]. Future efforts should focus on developing low energy, green pretreatment technologies that simultaneously separate high value lignin, and advancing in situ or biological detoxification techniques to reduce sugar loss and waste emissions [68]. Enzyme immobilization, formulation optimization, and recovery strategies should be employed to lower enzyme costs, while engineering microbial strains for simultaneous polysaccharide utilization should be developed [69,70]. Additionally, high solid fermentation reactors and mixing systems need to be optimized to address mass transfer and viscosity challenges, and regionalized raw material collection and storage supply chains should be established [71,72,73].

#### 2.1.2. Molasses

Molasses is a by-product of the sugar industry and is a waste product of molasses remaining after the removal of usable sugar from the raw molasses. Common molasses includes sugarcane molasses, sugar beet molasses, corn molasses, and soybean molasses. The main components of molasses are carbohydrates and water, with a high content of sucrose and reducing sugars, and a sucrose content of 40–60%. It is also rich in nutrients such as proteins, minerals, and vitamins. However, the composition of molasses is influenced by the sugar production process and the type of raw material. For example, sugarcane molasses is primarily composed of 35% sucrose, 12% fructose and glucose, ~9.6% ash (including inorganic salts like potassium, calcium, magnesium), plus small amounts of crude protein and bioflavonoids [74]. Soybean molasses contains 58–68% sugar (stachyose, raffinose, sucrose, and small amounts of monosaccharides). Minor components include 5–12% protein, 4–20% lipids, 3–9% ash, 6–15% soyasaponins, 0.8–2.5% isoflavones, and other organic compounds [75,76]. Compared to lignocellulosic biomass, molasses requires no hydrolysis or sophisticated pretreatment, making the fermentation process more economical and convenient [77]. Previous studies have shown that glucose, sucrose, molasses, and sugarcane juice hydrolysate were used as fermentation carbon sources, respectively, with the use of sucrose as a fermentation carbon source yielding higher lactic acid production [78], whereas molasses has an even greater advantage compared to sucrose because of the presence of calcium, nitrogen, and other components that provide better nutritional conditions for microorganisms [35]. As documented by Calabia et al. [79], a comparative analysis was conducted on fermentative D-lactic acid production by *L. delbrueckii*, with 13% sugarcane molasses, sugarcane juice and sugar beet juice serving as raw substrates that required no additional pretreatment steps. The results showed that sugarcane molasses produced D-lactic acid with a titer of 107 g/L, which was superior to that of beet juice and inferior to that of sugarcane juice, demonstrating that sugar cane molasses can be used for the production of D-lactic acid in an efficient manner. To further enhance the application efficacy of molasses, the researchers screened inexpensive nitrogen sources for synergistic fermentation with molasses for D-lactic acid production while pursuing high D-lactic acid titers. Liang et al. [36] achieved a D-lactic acid titer of 122.3 g/L via SSF using two-stage adaptive evolved *L. delbrueckii* S-NL31, with sugarcane molasses and soybean meal as fermentation substrates. Beitel et al. [35] optimized carbon and nitrogen substrates, achieving a D-lactic acid titer of 162 g/L via fed-batch cultivation of *L. delbrueckii* with untreated molasses and corn steep liquor. This result marks the highest documented D-lactic acid output for fermentation processes using molasses as the carbon source. In recent years, some researchers have used lactic acid producing bacteria other than *L. delbrueckii* in combination with beet molasses to produce D-lactic acid. Hu et al. [37] effectively increased the heterologous expression of *L-LDH* in *Zymomonas mobilis* through metabolic engineering and optimized fermentation conditions, and ultimately produced a D-lactic acid titer of 32.9 g/L using 20% sugar beet molasses. Genetic replacement of the *PDC1* gene in *Kluyveromyces marxianus* with a codon-optimized *D-LDH* gene from *Klebsiella oxytoca* enabled production of 66.26 g/L D-lactic acid from sugarcane molasses medium without supplementation of additional nutrients [38]. However, heavy metals, pigments, and suspended solids in molasses inhibit microbial growth and reduce target product accumulation [77]. To address this issue, existing studies have mostly employed single or combined pretreatment processes including hot-acid clarification, barium sulfate or calcium oxide precipitation, EDTA chelation, activated carbon adsorption, hydrolytic enzymes, and membrane separation can be used to remove the above impurities [80,81]. These additional processing steps increase the complexity and overall cost of the production process, offsetting the raw material price advantage of molasses and thereby limiting its broader industrial application. Therefore, constructing highly robust production strains through metabolic engineering that can directly utilize complex substrates eliminates the need for pretreatment and its associated costs, which is key to sustainable molasses-based D-lactic acid production. Molasses availability is severely restricted by the regional distribution and production scale of the sugar industry [82]. In addition, significant inter-batch compositional variability, coupled with high ash and heavy metal contents, increases the uncertainty of downstream processing, making it difficult to support long-term stable D-lactic acid production [83]. Therefore, developing low-cost impurity removal technologies, enhancing strain tolerance to high osmolarity and high impurity environments through adaptive evolution and metabolic engineering to enable direct fermentation of crude molasses. Additionally, establishing industrial-grade molasses grading quality control standards and blending regulation systems should be established, and multi-batch mixing can be adopted to stabilize component fluctuations [20,84,85,86] are needed.

#### 2.1.3. Starch

Crop and waste starches serve as alternative carbon feedstocks for lactic acid production, including starchy products derived from rice, corn, tapioca, and other crops utilizable by D-lactic acid producing strains [5]. Starch comprises two structural components, amylose and amylopectin. Starch must first be degraded via enzymatic hydrolysis into glucose and maltose to support subsequent fermentative conversion. Starch is hydrolyzed sequentially to oligosaccharides by α-amylase and to glucose by amyloglucosidase for LAB utilization [87]. Although some LAB, such as *Lactobacillus amylophilus*, *L. plantarum*, *Lactobacillus fermentum* and *Lactobacillus amylovorus*, partially hydrolyze starch [88], their native amylolytic activity is insufficient to completely convert starch into fermentable glucose, necessitating the addition of exogenous amylase. To overcome this limitation, Shinkawa et al. [89] engineered *L. plantarum* to secrete α-amylase, enabling the first direct fermentation of corn starch to D-lactic acid without external enzyme supplementation. The strain was modified with *L-LDH* gene deletion and heterologous α-amylase from *Streptococcus bovis*, and titer 73.4 g/L D-lactic acid after 48 h of fermentation. In addition to corn starch, brown rice and sweet potato starch can also be used for the production of D-lactic acid due to its richness in starch. Okano et al. [49] used brown rice as the substrate and the metabolic engineering strain *L. plantarum* NCIMB 8826, which knocked out the *L-LDH* gene, as the fermentation strain. After 10 repeated fermentation experiments, the D-lactic acid titer remained stable at 118.4 to 129.8 g/L. Cheng et al. [87] achieved a D-lactic acid titer of 37.06 g/L from 40 g/L sweet potato starch via *Lactococcus lactis* subsp. *lactis* with glucosidase-mediated hydrolysis. Genetically engineered *Lactobacillus amyloliquefaciens* can efficiently convert starch into D-lactic acid, while strains other than *L. amyloliquefaciens* have lower titers of starch for the production of D-lactic acid, which has greater room for improvement in the future. In short, starch is currently the dominant industrial carbon source for D-lactic acid production, featuring mature production technology and stable feedstock supply. However, high raw material costs and competition with food crops for land have limited its long-term development [90]. Further reduction in overall production costs can be achieved through process integration optimization, synergistic utilization of by-products, and the development of high temperature fermentation strains.

#### 2.1.4. Whey

Whey represents a predominant side stream from dairy manufacturing processes, with ample nutritional constituents such as lactose, proteins and water-soluble vitamins [5]. It is mostly allocated to animal feed production or discharged directly as waste effluent. However, whey exhibits high levels of biochemical oxygen demand and chemical oxygen demand. When discharged into natural water bodies, it depletes dissolved oxygen in the aquatic environment and causes severe environmental contamination [23]. Lactose in whey can be hydrolyzed by enzymes or acid into glucose and galactose for fermentation to produce D-lactic acid, and its use as a fermentation raw material can greatly reduce the pollution caused by it and realize the reuse of renewable resources. Liu et al. [50] used cheese whey powder as the main substrate for D-lactic acid production. Following neutral protease hydrolysis and minor yeast extract supplementation, batch fermentation with *L. bulgaricus* CGMCC 1.6970 attained a final titer of 113.18 g/L. Furthermore, with abundant lactose and protein components, whey permeate can serve as a high-quality dual source of carbon and nitrogen for D-lactic acid production. Sahoo et al. [51] achieved a D-lactic acid titer of 45 g/L through fed-batch fermentation using whey permeate as the sole substrate without exogenous nutrient supplementation. However, improving the conversion of lactose and galactose by strains is a difficult problem in the production of D-lactic acid using whey as a fermentation substrate. Lactose hydrolysis produces equal amounts of glucose and galactose. However, some LAB (e.g., *S. thermophilus*, *L. delbrueckii* subsp. *bulgaricus*, *L. delbrueckii* subsp. *lactis*, and *Lactobacillus acidophilus*) can produce β-galactosidase to break down lactose into glucose and galactose, but lack a complete Leloir galactose metabolic pathway, preventing further utilization of galactose and ultimately resulting in the waste of approximately 50% of the fermentable carbon source [91]. The problem can be solved by modifying the strains using genetic engineering techniques or by using strain co-culture. Mukherjee et al. [92] successfully completed the Leloir pathway by heterologously expressing the *galK* and *galT* genes from *Lactobacillus helveticus* CNRZ32 in *L. delbrueckii* subsp. *bulgaricus* VI104, enabling it to produce D-lactic acid from galactose. However, *L. delbrueckii* can convert lactose into D-lactic acid and galactose, but cannot convert galactose; on the other hand, *L. lactis* can effectively convert galactose, but it predominantly produces L-lactic acid. Therefore, Sahoo et al. [51] engineered *L. lactis* TSG3 by knocking out its *L-LDH* gene to eliminate L-lactic acid production. They then co-cultured this engineered strain with *L. delbrueckii* to enhance the conversion of lactose and galactose into D-lactic acid. This strategy increased D-lactic acid production from whey. Since whey is a dual source of carbon and nitrogen, improving the ability of strains to metabolize lactose and galactose is essential for D-lactic acid titers in whey fermentations, but it still faces various challenges. Raw material composition varies greatly. Lactose, ash, and salt ion contents are affected by milk source, batch, processing, and season. Moreover, strains have low metabolic efficiency for galactose from lactose degradation in whey. Relying only on the strain’s endogenous β-galactosidase cannot achieve sufficient hydrolysis, so lactase must be added to assist substrate breakdown. Meanwhile, the rich nutrients of whey are prone to the growth of miscellaneous microorganisms, which increases the process complexity and the risk of fermentation failure [51,92]. Metabolic engineering, adaptive evolution and strain optimization can enhance strains’ galactose metabolism and osmotic tolerance, while immobilized lactase technology can improve lactose hydrolysis, develop a cascade utilization process to recover whey protein, and produce D-lactic acid through integrated fermentation [20,86].

#### 2.1.5. Algae

Algae are photosynthetic organisms that can grow in a variety of habitats such as lakes, rivers, and oceans, and can usually be categorized into macroalgae (seaweeds) and microalgae [93]. Algae are rich in glucose, galactose, mannose, cellulose and other sugars, which can be used for D-lactic acid production because of their high polysaccharide content and their ability to be produced rapidly and on a large scale [94]. The process of D-lactic acid production from algae usually consists of five steps: milling, pretreatment, hydrolysis, fermentation and distillation. In the hydrolysis step most algae bioproduction uses enzymatic hydrolysis, which increases the surface area of algae action and improves the conversion rate of algae into fermentable sugars [95]. Nguyen et al. [52] evaluated enzymatic hydrolysis of *Hydrodictyon reticulatum* and D-lactic acid fermentation by *Lactobacillus coryniformis* subsp. *torquens* ATCC 25600. α-amylase was dispensable, and optimal hydrolysis was obtained with cellobiase and amyloglucosidase at 50% of the dosage used for L-lactic acid production by *Lactobacillus paracasei* LA104. D-lactic acid titer of 36.6 g/L was achieved after 36 h fermentation in microalgal dry matter medium supplemented with yeast extract and peptone. Niccolai et al. [53] used lyophilised biomass *Arthrospira platensis* F&M-C256 as the sole substrate, and *L. plantarum* ATCC 8014 reached a lactic acid titer of 3.7 g/L after 48 h. Qiu et al. [21] reported that the acid hydrolyzed solution of *Gelidium amansii* mainly contains galactose, glucose, and a small amount of xylose. After pretreatment and biodegradation detoxification, SSCF by *P. acidilactici* resulted in a record high of D-lactic acid titer (41.4 g/L). Overall, algae, a third-generation biomass feedstock, has great long-term potential, but most current research is still at the small-scale laboratory stage, with multiple challenges for practical applications [96]. Current studies show that the titers of D-lactic acid produced by algal substrate fermentation are generally below 50 g/L, significantly lower than the over 100 g/L achieved with lignocellulosic substrates [97]. The underlying reason is that the solid loading of algal biomass is only approximately 10% (*w*/*w*), which is substantially lower than the 30% (*w*/*w*) solid loading typical of lignocellulosic biomass. Glycosidases have low commercialization levels, while algae carry contaminants and algal toxins, making pollution control and quality assurance difficult [98]. Additionally, the main hydrolysis products of algal biomass are galactose and mannitol, both of which are difficult for commonly used microbial strains to utilize efficiently [21]. When combined with CCR and mass transfer limitations induced by gel like by-products generated from incomplete pretreatment, these factors collectively constrain the accumulation of target fermentation products [99,100,101]. Therefore, develop low-cost harvesting technologies and mild cell disruption processes to cut raw material pretreatment costs. Develop high efficiency degrading enzymes to reduce enzyme preparation costs, while alleviating mass transfer limitations caused by increased viscosity of enzymatic hydrolysates [102]. Modify strains’ galactose and mannitol metabolic pathways via metabolic engineering to realize simultaneous and efficient utilization of algae derived mixed carbon sources [103].

#### 2.1.6. Other Alternative Carbon Sources

Apart from the alternative carbon sources outlined above, several renewable feedstocks work as viable carbon substrates for D-lactic acid fermentation. For example, crude glycerol from the biodiesel industry is one option [5]; it is a high-quality carbon source for industrial microbial utilization, with abundant source, high reducibility and non-toxicity [104]. Wang et al. [54] adapted the strain for laboratory evolution in crude glycerol by optimizing the glycerol isomerization pathway and D-lactic acid production pathway of *E. coli* with a batch fermentation D-lactic acid titer of 100 g/L. Inulin is a semi-wild plant compound which contains 75.88% water, 12.46% sugar and 1.58% protein, as well as fat and trace elements. Inulin is a naturally occurring polysaccharide that stores energy and contains 80% of the total sugar in the tubers [105], and after enzymatic or acid hydrolysis the raw material is rich in fructose, glucose, and other fermentable raw materials. Inulinase is the most commonly used hydrolase for the hydrolysis of inulin, with fewer enzymatic by-products and milder reactions than acid hydrolysis. The production of D-lactic acid from inulin by metabolic engineering techniques has been investigated; Bae et al. [56] constructed a metabolically engineered *K. marxianus* with *L-LDH* gene deletion, and produced D-lactic acid with high optical purity using inulin as the carbon source. When using inulin tubers containing 140 g/L inulin as the substrate, the D-lactic acid titer reached 122 g/L. Xu et al. [55] adopted a SSF strategy to produce D-lactic acid from inulin with *L. bulgaricus* CGMCC 1.6970, and attained a D-lactic acid titer of 123.6 g/L. These findings indicate that glycerol and inulin are favorable carbon substrates for D-lactic acid fermentation, and can support higher product titers. Overall, crude glycerol and inulin represent underutilized yet high-potential feedstocks for D-lactic acid biosynthesis. Further advances in strain tolerance engineering and in situ process integration will be critical to unlocking their full economic and environmental value for large-scale sustainable production.

### 2.2. Production of D-Lactic Acid Using Renewable Resources as a Nitrogen Source

As heterotrophic microorganisms, the majority of lactic acid-producing bacteria require exogenous supplementation of growth factors such as vitamins and amino acids to support normal growth and biosynthetic processes, in addition to providing a suitable carbon source for microbial fermentation. Yeast extract serves as an effective complex nutrient that supports bacterial strain growth and finds extensive application in lactic acid fermentation. Nevertheless, according to the efficiency analysis of lactic acid production, the cost of using yeast extract as a source of fermentation nitrogen accounts for 38% of the total cost, so there is a need to find cheap nutrients to replace yeast extract [14]. Table 3 summarizes recent studies on the fermentation of D-lactic acid using cottonseed, soybean meal, corn steep liquor, and peanut meal as alternative nitrogen sources to yeast extract.

#### 2.2.1. Cottonseed Meal

Cottonseed is the seed of the cotton crop, consisting of a hard outer shell and an inner kernel. The kernel contains 35–45% oil. Cottonseed meal refers to the residual by-product generated after oil extraction from cottonseed. With a protein content of 30–50%, this meal serves as a high-quality nitrogen source for microbial fermentation [111]. Cheap renewable resources as nitrogen sources often require the addition of a certain amount of yeast extract to meet microbial growth, while cottonseed meal can be used as the sole nitrogen source to produce D-lactic acid. Li et al. [106] obtained a D-lactic acid titer of 144.4 g/L via fermentation by *S. laevolacticus* DSM442, with cottonseed meal serving as the sole nitrogen source. Bai et al. [40] further confirmed that *S. inulinus* YBS1-5 can also utilize cottonseed meal as the sole nitrogen source for D-lactic acid synthesis. They additionally optimized the fermentation carbon source by adopting corncob residue as the carbon substrate, and obtained a final D-lactic acid titer of 107.2 g/L, demonstrating the feasibility of synergistic utilization of cottonseed meal and lignocellulose. Compared with yeast extract, cottonseed meal exhibits an unbalanced amino acid composition, with relatively low contents of essential amino acids such as lysine and methionine [112]. Protein in cottonseed meal mainly exists in large molecular forms, requiring extracellular proteases secreted by microorganisms to hydrolyze them into small peptides and amino acids, which may lead to an extended lag phase during the early stages of fermentation. To improve peptide availability, enzymatic hydrolysis is often required [113]. Cottonseed meal contains the anti-nutritional factor gossypol, which inhibits the fermentation of strains, potentially reducing fermentation rate and titer. Moreover, residual gossypol may compromise the safety of downstream products [114,115]. Studies have shown that a series of processes can remove gossypol from cottonseed meal. However, detoxification and hydrolysis raise energy, equipment and operational costs. Meanwhile, concentrated raw material supply, high storage and transportation costs, and mold contamination further limit its large-scale application. Therefore, future research on cottonseed meal should not only improve D-lactic acid titers under laboratory conditions, but also establish a raw material grading and blending system to stabilize batch variations, and address amino acid nutritional deficiencies via optimized nitrogen source combinations. Additionally, researchers should further develop low-loss green detoxification processes and engineer high-yield gossypol-resistant strains through metabolic engineering and adaptive evolution.

#### 2.2.2. Soybean Meal

Soybean meal is a by-product of soybean oil extraction from soybeans, containing 44–49% crude protein and 5.2–9.1% crude ash, which can be used as animal feed, protein supplements or biodiesel production [116]. However, soybean protein mainly consists of tightly structured glycinin and β-conglycinin, with relatively low levels of free amino acids and small peptides lower than those in yeast extract. Most microbial strains cannot directly and efficiently utilize these components, requiring prior enzymatic or acid hydrolysis to release active nitrogenous compounds. The degree of hydrolysis is a key factor determining fermentation efficiency [117]. In addition, soybean meal is rich in lysine but relatively low in methionine and cysteine. It also contains anti-nutritional factors such as trypsin inhibitors, phytic acid, and soybean lectins, which can inhibit microbial metabolic enzyme activity and reduce the bioavailability of mineral elements. Thus, moderate heat treatment is required to eliminate most of these negative effects [118]. Conducting research on the production of D-lactic acid, Zhang et al. [31] achieved a D-lactic acid titer of 61.4 g/L via fed-batch fermentation using corn stover and soybean meal extract. To further enhance D-lactic acid production with soybean meal as the fermentation nitrogen source, Nguyen et al. [47] performed SSCF with *L. coryniformis* subsp. *torquens* ATCC 25600. They used spent turmeric biomass derived from turmeric extraction and soybean meal as fermentation substrates, and obtained a maximum D-lactic acid titer of 89.06 g/L at the optimal soybean meal dosage of 15 g/L. When soybean meal serves as the fermentation nitrogen source, the final D-lactic acid titer is likely associated with the strain’s utilization efficiency of different carbon substrates. Liang et al. [36] adopted sugarcane molasses and soybean meal, two common agro-industrial wastes, as fermentation feedstocks. By coupling simultaneous enzymatic hydrolysis of soybean meal with a fed-batch fermentation strategy, they achieved a D-lactic acid titer of 112.3 g/L. The above studies show that soybean meal combined with molasses, an inexpensive carbon source, as a substrate for D-lactic acid fermentation was able to achieve higher D-lactic acid titers than in combination with lignocellulose.

#### 2.2.3. Corn Steep Liquor

Corn steep liquor is a by-product produced by the corn starch industry, which contains vitamins, amino acids and other nutrients, and is a cheap source of nitrogen that can be utilized for the production of D-lactic acid by fermentation [35]. Compared to yeast extract, corn steep liquor has a very low raw material cost, and its nutritional components are mostly in small molecular forms that can be directly utilized, eliminating the need for additional hydrolysis processes. However, raw corn steep liquor contains a high concentration of L-lactic acid, which would compromise the optical purity of the final D-lactic acid product. To address this issue, Okano et al. [107] heterologously expressed L-lactate oxidase from *Enterococcus* subsp. NBRC 3427 in *L. plantarum* KOLP7. This modification successfully eliminated endogenous L-lactic acid present in the corn steep liquor, thereby preserving the optical purity of the D-lactic acid produced. Using corn steep liquor and lignocellulosic hydrolysate as fermentation substrates, the engineered strain produced D-lactic acid with an optical purity of 99.99% and a titer of 41.7 g/L. Furthermore, corn steep liquor can be applied together with molasses, as combined feedstocks for D-lactic acid fermentation. Beitel et al. [35] used molasses, an agricultural waste, and corn steep liquor as fermentation feedstock, which was effectively converted to D-lactic acid by *L. delbrueckii*, with a D-lactic acid titer of up to 162 g/L. In a word, corn steep liquor can be used as a good nitrogen source for D-lactic acid fermentation, which is used as a source of nitrogen for fermentation, and the level of D-lactic acid titer might depend on the effectiveness of the strain in converting the fermentation carbon source.

#### 2.2.4. Peanut Meal

Peanut meal is a by-product of peanut oil extraction from peanut kernels, which contains proteins, trace elements and a small amount of peanut oil, which can be used as a nutrient for D-lactic acid fermentation [110]. Wang et al. [110] evaluated the effects of seven nitrogen sources, including yeast extract, beef paste, soybean peptone, peanut meal, soybean meal, corn syrup, and ammonium sulfate, on the production of D-lactic acid by *Sporolactobacillus* sp. CASD. The results showed that peanut meal produced the highest titer of D-lactic acid, reaching 207 g/L with an average productivity of 3.8 g/L/h using 40 g/L peanut meal in a 30 L fed-batch fermentation process. High D-lactic acid titers were similarly achieved using genetic engineering breeding techniques combined with peanut meal as a fermentation nitrogen source. Assavasirijinda et al. [109] constructed an engineered *Bacillus alkaliphilic* strain via genetic modification. They knocked out the native *L-LDH* gene and introduced the *D-LDH* gene from *L. delbrueckii* to enhance D-lactic acid production. With peanut meal as the nitrogen source, the strain achieved a D-lactic acid titer of 143.99 g/L through fed-batch fermentation. These results confirm the effectiveness of peanut meal as a nitrogen substrate for D-lactic acid fermentation. However, the protein in untreated peanut meal is large-molecular and has unbalanced amino acid composition. Unlike yeast extract, peanut meal is easily contaminated by Aspergillus flavus, which raises both product safety risks and costs for raw material quality control and detoxification. Thus, future research should focus on developing mild efficient detoxification technologies, building a fermentation system combining peanut meal with low-cost nitrogen sources, and modifying microbial strains to improve toxin and high oil environment tolerance.

## 3. Conclusions and Future Perspective

D-lactic acid is a key monomer for PLA production, and its production cost remains the main barrier to PLA’s widespread application. Among the aforementioned carbon sources, molasses and lignocellulose both hold significant industrial potential. The sugars in molasses can be directly utilized by most microorganisms without complex pretreatment, and when combined with inexpensive nitrogen sources such as corn steep liquor or cottonseed meal, they meet the nutritional requirements of microbial strains, offering strong industrial competitiveness. However, their supply is limited by production regions and capacity, making them vulnerable to risks of raw material shortages and price fluctuations. Lignocellulosic feedstocks are abundant and widely distributed, making them suitable for large-scale applications. However, they require complex pretreatment and enzymatic hydrolysis processes, which are costly and generate fermentation inhibitors, leaving a significant technological gap for industrial-scale production. Currently, molasses is an economically viable carbon source in industry, while lignocellulose is alternative feedstock with long-term potential. In the future, starch feedstocks are likely to remain the dominant carbon source for industrial D-lactic acid production, while diversified carbon sources will be developed regionally in accordance with local industries. For nitrogen supply, cottonseed meal, soybean meal, peanut meal, and corn steep liquor have all demonstrated high D-lactic acid titers when combined with inexpensive carbon sources, with yields depending largely on the strain’s ability to convert the chosen carbon substrate. Corn steep liquor is a by-product of processed corn, with a lower cost than yeast extract and peptone. It is rich in soluble proteins, free amino acids, biotin, and other natural growth factors, making it a high-cost performance nitrogen source suitable for large-scale production. In conclusion, the utilization of these renewable resources has seen its economic viability compromised by additional pretreatment processes and other contributing factors. Therefore, in the future, the comprehensive cost of carbon and nitrogen sources for D-lactic acid production can be systematically reduced by optimizing the pretreatment of carbon and nitrogen sources and fermentation processes, breeding strains with high substrate conversion rate and stress resistance, improving nutrient utilization efficiency via optimizing the ratio of composite carbon and nitrogen sources, and developing low-cost detoxification technologies.

## Figures and Tables

**Table 1:** Comparison of D-lactic acid fermentation patterns.

Fermentation Mode	Advantages	Disadvantages	Industrial Relevance	Reference
Batch fermentation	Low risk of contamination and simple operation	Long fermentation time, low lactic acid titer, product inhibition by initial substrate concentration	Only applied for small-batch trial production, raw material screening and strain domestication	[11]
Fed-batch fermentation	High lactic acid titer and productivity	High risk of contamination and complexity of operation	Balances production performance and operational difficulty, highest industrial adoption rate	[12]
Continuous fermentation	High lactic acid titer and productivity	High equipment requirements, susceptible to cell loss and strain mutation	Hard to maintain long-term stable D-lactic acid optical purity, not widely popularized	[13]
Cell-recycle fermentation	High cell density fermentation, high lactic acid titer, high productivity	Complex process, high economic cost, obstacles to cell spreading	Still confined to pilot scale verification	[14]

**Table 3:** Research progress of renewable resources for nitrogen source in D-lactic acid fermentation.

Substrate	Strains	Fermentation Mode	Titer (g/L)	Productivity (g/L/h)	Optical Purity (%)	Reference
Cottonseed meal	*S. inulinus* YBS1-5	Fed-batch (7 L)	107.2	1.19	99.2	[40]
Cottonseed	*S. laevolacticus* DSM442	Fed-batch (5 L)	144.4	4.13	99.3	[106]
Soybean meal	*L. delbrueckii*	Fed-batch (7.5 L)	112.3	2.4	99.6	[36]
Soybean meal	*L. plantarum* NCIMB 8826	Fed-batch (2 L)	61.4	0.32	99	[31]
Corn steep liquor	*L. delbrueckii* ssp. *delbrueckii*	- (100 mL)	-	2.35	-	[44]
Corn steep liquor	*L. delbrueckii*	Fed-batch (750 mL)	162	3.37	97.66	[35]
Corn steep liquor	*L. plantarum* KOLP7	-	41.7	-	99.99	[107]
Sugarcane bagasse	*P. acidilactici* XH11	SSCF	57.0	0.79		[28]
Peanut meal	*S. nakayamae*	-	112.93	1.57	98.75	[108]
Peanut meal	*B. alkaliphilic*	Fed-batch (1.5 L)	143.99	1.674	99.85	[109]
Peanut meal	*Sporolactobacillus* sp. CASD	Fed-batch (30 L)	207	3.8	99.3	[110]

“-”: Indicates that the relevant content was not found.

## Data Availability

No new data were created or analyzed in this study. Data sharing is not applicable to this article.

## References

[1] de França J.O.C., da Silva Valadares D., Paiva M.F., Dias S.C.L., Dias J.A. (2022). Polymers based on PLA from synthesis using D,L-lactic acid (or racemic lactide) and some biomedical applications: A short review. Polymers.

[2] Ahmadi H., Looijmans S.F.S.P., van Maris M.P.F.H.L., Schmit P., Maraghechi S., Anderson P.D., Cardinaels R. (2025). Formation of PLA stereocomplex crystals through homorecrystallization and mesophase growth mechanisms. Macromolecules.

[3] Augustiniene E., Valanciene E., Matulis P., Syrpas M., Jonuskiene I., Malys N. (2022). Bioproduction of L- and D-lactic acids: Advances and trends in microbial strain application and engineering. Crit. Rev. Biotechnol..

[4] Yan Y., Xu R., Li X., Yao Z., Zhang H., Li H., Chen W. (2022). Unexpected immunoregulation effects of D-lactate, different from L-lactate. Food Agric. Immunol..

[5] Abedi E., Hashemi S.M.B. (2020). Lactic acid production—Producing microorganisms and substrates sources-state of art. Heliyon.

[6] Sae-Tang K., Bumrungtham P., Mhuantong W., Champreda V., Tanapongpipat S., Zhao X.-Q., Liu C.-G., Runguphan W. (2023). Engineering flocculation for improved tolerance and production of D-lactic acid in *Pichia pastoris*. J. Fungi.

[7] Wu B., Yu Q., Zheng S., Pedroso M.M., Guddat L.W., He B., Schenk G. (2019). Relative catalytic efficiencies and transcript levels of three D- and two L-lactate dehydrogenases for optically pure D-lactate production in *Sporolactobacillus inulinus*. Microbiologyopen.

[8] Eş I., Mousavi Khaneghah A., Barba F.J., Saraiva J.A., Sant’Ana A.S., Hashemi S.M.B. (2018). Recent advancements in lactic acid production—A review. Food Res. Int..

[9] Wang Y., Tashiro Y., Sonomoto K. (2015). Fermentative production of lactic acid from renewable materials: Recent achievements, prospects, and limits. J. Biosci. Bioeng..

[10] Rawoof S.A.A., Kumar P.S., Vo D.-V.N., Devaraj K., Mani Y., Devaraj T., Subramanian S. (2021). Production of optically pure lactic acid by microbial fermentation: A review. Environ. Chem. Lett..

[11] Fu X., Wang Y., Wang J., Garza E., Manow R., Zhou S. (2017). Semi-industrial scale (30 m(3)) fed-batch fermentation for the production of D-lactate by *Escherichia coli* strain HBUT-D15. J. Ind. Microbiol. Biotechnol..

[12] Michelz Beitel S., Fontes Coelho L., Sass D.C., Contiero J. (2017). Environmentally friendly production of D(-) lactic acid by *Sporolactobacillus nakayamae*: Investigation of fermentation parameters and fed-batch strategies. Int. J. Microbiol..

[13] Aso Y., Tsubaki M., Dang Long B.H., Murakami R., Nagata K., Okano H., Phuong Dung N.T., Ohara H. (2019). Continuous production of D-lactic acid from cellobiose in cell recycle fermentation using β-glucosidase-displaying *Escherichia coli*. J. Biosci. Bioeng..

[14] Ma K., Cui Y., Zhao K., Yang Y., Wang Y., Hu G., He M. (2022). D-lactic acid production from agricultural residues by membrane integrated continuous fermentation coupled with B vitamin supplementation. Biotechnol. Biofuels Bioprod..

[15] Smerilli M., Neureiter M., Wurz S., Haas C., Fruehauf S., Fuchs W. (2015). Direct fermentation of potato starch and potato residues to lactic acid by *Geobacillus stearothermophilus* under non-sterile conditions. J. Chem. Technol. Biotechnol..

[16] Qian X., Yan W., Zhang W., Dong W., Ma J., Ochsenreither K., Jiang M., Xin F. (2019). Current status and perspectives of 2-phenylethanol production through biological processes. Crit. Rev. Biotechnol..

[17] Mitri S., Koubaa M., Maroun R.G., Rossignol T., Nicaud J.M., Louka N. (2022). Bioproduction of 2-phenylethanol through yeast fermentation on synthetic media and on agro-industrial waste and by-products: A review. Foods.

[18] Bai Z., Gao Z., He B., Wu B. (2015). Effect of lignocellulose-derived inhibitors on the growth and D-lactic acid production of *Sporolactobacillus inulinus* YBS1-5. Bioprocess Biosyst. Eng..

[19] Sun Y., Xu Z., Zheng Y., Zhou J., Xiu Z. (2019). Efficient production of lactic acid from sugarcane molasses by a newly microbial consortium CEE-DL15. Process Biochem..

[20] Mukherjee P., Raj N., Sivaprakasam S. (2023). Harnessing valorization potential of whey permeate for D-lactic acid production using lactic acid bacteria. Biomass Convers. Biorefinery.

[21] Qiu Z., Wang G., Shao W., Cao L., Tan H., Shao S., Jin C., Xia J., He J., Liu X. (2024). Third-generation D-lactic acid production using red macroalgae *Gelidium amansii* by co-fermentation of galactose, glucose and xylose. Bioresour. Technol..

[22] Huang J., Wang J., Liu S. (2023). Advanced fermentation techniques for lactic acid production from agricultural waste. Fermentation.

[23] Pires A.F., Marnotes N.G., Rubio O.D., Garcia A.C., Pereira C.D. (2021). Dairy by-products: A review on the valorization of whey and second cheese whey. Foods.

[24] Tian X., Chen H., Liu H., Chen J. (2021). Recent advances in lactic acid production by lactic acid bacteria. Appl. Biochem. Biotechnol..

[25] Zaini N., Chatzifragkou A., Charalampopoulos D. (2019). Microbial production of D-lactic acid from dried distiller’s grains with solubles. Eng. Life Sci..

[26] Yankov D. (2022). Fermentative lactic acid production from lignocellulosic feedstocks: From source to purified product. Front. Chem..

[27] He N., Chen M., Qiu Z., Fang C., Lidén G., Liu X., Zhang B., Bao J. (2023). Simultaneous and rate-coordinated conversion of lignocellulose derived glucose, xylose, arabinose, mannose, and galactose into D-lactic acid production facilitates D-lactide synthesis. Bioresour. Technol..

[28] Qiu Z., Han X., Fu A., Jiang Y., Zhang W., Jin C., Li D., Xia J., He J., Deng Y. (2023). Enhanced cellulosic D-lactic acid production from sugarcane bagasse by pre-fermentation of water-soluble carbohydrates before acid pretreatment. Bioresour. Technol..

[29] Zhang Z., Li Y., Zhang J., Peng N., Liang Y., Zhao S. (2020). High-titer lactic acid production by *Pediococcus acidilactici* PA204 from corn stover through fed-batch simultaneous saccharification and fermentation. Microorganisms.

[30] Qiu Z., Gao Q., Bao J. (2017). Constructing xylose-assimilating pathways in *Pediococcus acidilactici* for high titer D-lactic acid fermentation from corn stover feedstock. Bioresour. Technol..

[31] Zhang Y., Vadlani P.V., Kumar A., Hardwidge P.R., Govind R., Tanaka T., Kondo A. (2016). Enhanced D-lactic acid production from renewable resources using engineered *Lactobacillus plantarum*. Appl. Microbiol. Biotechnol..

[32] Yi X., Zhang P., Sun J., Tu Y., Gao Q., Zhang J., Bao J. (2016). Engineering wild-type robust *Pediococcus acidilactici* strain for high titer L- and D-lactic acid production from corn stover feedstock. J. Biotechnol..

[33] Wang X., Wang G., Yu X., Chen H., Sun Y., Chen G. (2017). Pretreatment of corn stover by solid acid for D-lactic acid fermentation. Bioresour. Technol..

[34] Qiu Z., Han X., He J., Jiang Y., Wang G., Wang Z., Liu X., Xia J., Xu N., He A. (2022). One-pot D-lactic acid production using undetoxified acid-pretreated corncob slurry by an adapted *Pediococcus acidilactici*. Bioresour. Technol..

[35] Beitel S.M., Coelho L.F., Contiero J. (2020). Efficient conversion of agroindustrial waste into D(-) lactic acid by *Lactobacillus delbrueckii* using fed-batch fermentation. BioMed Res. Int..

[36] Liang S., Jiang W., Song Y., Zhou S.F. (2020). Improvement and metabolomics-based analysis of D-lactic acid production from agro-industrial wastes by *Lactobacillus delbrueckii* submitted to adaptive laboratory evolution. J. Agric. Food Chem..

[37] Hu M., Bao W., Peng Q., Hu W., Yang X., Xiang Y., Yan X., Li M., Xu P., He Q. (2023). Metabolic engineering of *Zymomonas mobilis* for co-production of D-lactic acid and ethanol using waste feedstocks of molasses and corncob residue hydrolysate. Front Bioeng. Biotechnol..

[38] Gosalawit C., Khunnonkwao P., Jantama K. (2023). Genome engineering of *Kluyveromyces marxianus* for high D-( −)-lactic acid production under low pH conditions. Appl. Microbiol. Biotechnol..

[39] Ting Z., Dong L., Hengfei R., Xinchi S., Nan Z., Yong C. (2014). D-lactic acid production by *Sporolactobacillus inulinus* Y2-8 immobilized in fibrous bed bioreactor using corn flour hydrolyzate. J. Microbiol. Biotechnol..

[40] Bai Z., Gao Z., Sun J., Wu B., He B. (2016). D-lactic acid production by *Sporolactobacillus inulinus* YBS1-5 with simultaneous utilization of cottonseed meal and corncob residue. Bioresour. Technol..

[41] de Oliveira Moraes A., Ramirez N.I.B., Pereira N. (2016). Evaluation of the fermentation potential of pulp mill residue to produce D(−)-lactic acid by separate hydrolysis and fermentation using *Lactobacillus coryniformis* subsp. *torquens*. Appl. Biochem. Biotechnol..

[42] Hama S., Mizuno S., Kihara M., Tanaka T., Ogino C., Noda H., Kondo A. (2015). Production of D-lactic acid from hardwood pulp by mechanical milling followed by simultaneous saccharification and fermentation using metabolically engineered *Lactobacillus plantarum*. Bioresour. Technol..

[43] Alexandri M., Hübner D., Schneider R., Fröhling A., Venus J. (2022). Towards efficient production of highly optically pure D-lactic acid from lignocellulosic hydrolysates using newly isolated lactic acid bacteria. New Biotechnol..

[44] de la Torre I., Ladero M., Santos V.E. (2018). Production of D-lactic acid by *Lactobacillus delbrueckii* ssp. *delbrueckii* from orange peel waste: Techno-economical assessment of nitrogen sources. Appl. Microbiol. Biotechnol..

[45] Balakrishnan R., Reddy Tadi S.R., Sivaprakasam S., Rajaram S. (2018). Optimization of acid and enzymatic hydrolysis of kodo millet (*Paspalum scrobiculatum*) bran residue to obtain fermentable sugars for the production of optically pure D (−) lactic acid. Ind. Crops Prod..

[46] Reddy Tadi S.R., E.V.R. A., Limaye A.M., Sivaprakasam S. (2017). Enhanced production of optically pure D (–) lactic acid from nutritionally rich *Borassus flabellifer* sugar and whey protein hydrolysate based–fermentation medium. Biotechnol. Appl. Biochem..

[47] Nguyen C.M., Kim J.-S., Nguyen T.N., Kim S.K., Choi G.J., Choi Y.H., Jang K.S., Kim J.-C. (2013). Production of L- and D-lactic acid from waste *Curcuma longa* biomass through simultaneous saccharification and cofermentation. Bioresour. Technol..

[48] Okano K., Zhang Q., Shinkawa S., Yoshida S., Tanaka T., Fukuda H., Kondo A. (2009). Efficient production of optically pure D-lactic acid from raw corn starch by using a genetically modified L-lactate dehydrogenase gene-deficient and α-amylase-secreting *Lactobacillus plantarum* Strain. Appl. Environ. Microbiol..

[49] Okano K., Hama S., Kihara M., Noda H., Tanaka T., Kondo A. (2017). Production of optically pure D-lactic acid from brown rice using metabolically engineered *Lactobacillus plantarum*. Appl. Microbiol. Biotechnol..

[50] Liu P., Zheng Z., Xu Q., Qian Z., Liu J., Ouyang J. (2018). Valorization of dairy waste for enhanced D-lactic acid production at low cost. Process. Biochem..

[51] Sahoo T.K., Jayaraman G. (2019). Co-culture of *Lactobacillus delbrueckii* and engineered *Lactococcus lactis* enhances stoichiometric yield of D-lactic acid from whey permeate. Appl. Microbiol. Biotechnol..

[52] Nguyen C.M., Kim J.-S., Song J.K., Choi G.J., Choi Y.H., Jang K.S., Kim J.-C. (2012). D-lactic acid production from dry biomass of *Hydrodictyon reticulatum* by simultaneous saccharification and co-fermentation using *Lactobacillus coryniformis* subsp. *Torquens*. Biotechnol. Lett..

[53] Niccolai A., Shannon E., Abu-Ghannam N., Biondi N., Rodolfi L., Tredici M.R. (2019). Lactic acid fermentation of *Arthrospira platensis* (spirulina) biomass for probiotic-based products. J. Appl. Phycol..

[54] Wang Y.D., Liao J.Y., Chiang C.J., Chao Y.P. (2019). A simple strategy to effectively produce D-lactate in crude glycerol-utilizing *Escherichia coli*. Biotechnol. Biofuels.

[55] Xu Q., Zang Y., Zhou J., Liu P., Li X., Yong Q., Ouyang J. (2016). Highly efficient production of D-lactic acid from chicory-derived inulin by *Lactobacillus bulgaricus*. Bioprocess Biosyst. Eng..

[56] Bae J.H., Kim H.J., Kim M.J., Sung B.H., Jeon J.H., Kim H.S., Jin Y.S., Kweon D.H., Sohn J.H. (2018). Direct fermentation of Jerusalem artichoke tuber powder for production of L-lactic acid and D-lactic acid by metabolically engineered *Kluyveromyces marxianus*. J. Biotechnol..

[57] Han T., Wang Y., Zhang B., Bao J. (2026). Fast fluidization of dry acid pretreated wheat straw and consequent continuous bioconversions for L-lactic acid production. Bioresour. Technol..

[58] Fan J., Lu Y., An N., Zhu W.B., Li M., Gao M., Wang X., Wu C., Wang Y. (2025). Pretreatment technologies for lignocellulosic biomass: Research progress, mechanisms, and prospects. BioResources.

[59] Fernandes M.C., Alves-Ferreira J., Duarte L.C., Pereira H., Carvalheiro F., Martínez A. (2023). D-lactic acid production from hydrothermally pretreated, alkali delignified and enzymatically saccharified rockrose with the metabolic engineered *Escherichia coli* strain JU15. Biomass Convers. Biorefinery.

[60] Gugel I., Marchetti F., Costa S., Baldini E., Vertuani S., Manfredini S. (2025). Efficient downstream processing of second-generation lactic acid from lignocellulosic waste using aqueous two-phase extraction. Bioresour. Bioprocess..

[61] Wang X., Khushk I., Xiao Y., Gao Q., Bao J. (2018). Tolerance improvement of *Corynebacterium glutamicum* on lignocellulose derived inhibitors by adaptive evolution. Appl. Microbiol. Biotechnol..

[62] Hu W., Zhou L., Chen J.H. (2022). Conversion sweet sorghum biomass to produce value-added products. Biotechnol. Biofuels Bioprod..

[63] Nair A., Sarma S.J. (2021). The impact of carbon and nitrogen catabolite repression in microorganisms. Microbiol. Res..

[64] Lu H., Zhao X., Wang Y., Ding X., Wang J., Garza E., Manow R., Iverson A., Zhou S. (2016). Enhancement of D-lactic acid production from a mixed glucose and xylose substrate by the *Escherichia coli* strain JH15 devoid of the glucose effect. BMC Biotechnol..

[65] Sulis D.B., Lavoine N., Sederoff H., Jiang X., Marques B.M., Lan K., Cofre-Vega C., Barrangou R., Wang J.P. (2025). Advances in lignocellulosic feedstocks for bioenergy and bioproducts. Nat. Commun..

[66] Wu R., Yang J., Jiang Y., Xin F. (2025). Advances and prospects for lactic acid production from lignocellulose. Enzym. Microb. Technol..

[67] Waldschitz D., Bartlechner J., Karner E.M., Spadiut O., Jakubek S., Kager J. (2025). Addressing raw material variation: Maintaining a steady-state during cultivation by blending of lignocellulosic feed streams. Biochem. Eng. J..

[68] Rajapakse H., Raviadaran R., Chandran D. (2025). Deep eutectic solvents for pretreatment of lignocellulosic biomass: Towards circular bioeconomy. Results Eng..

[69] Joo H., Lee H.-Y., Sim H.-Y., Jung C.-D., Choi J.-H., Lee J.-C., Myung S. (2026). Sustainable removal of phenolic impurities from lignocellulosic-biomass-derived sugars using immobilized laccase and membrane filtration. Sep. Purif. Technol..

[70] Nogueira C.d.C., Padilha C.E.d.A., Dantas J.M.d.M., Medeiros F.G.M.d., Guilherme A.d.A., Souza D.F.d.S., Santos E.S.d. (2021). In-situ detoxification strategies to boost bioalcohol production from lignocellulosic biomass. Renew. Energy.

[71] Jeong D., Ye S., Park H., Kim S.R. (2020). Simultaneous fermentation of galacturonic acid and five-carbon sugars by engineered *Saccharomyces cerevisiae*. Bioresour. Technol..

[72] Roni M.S., Thompson D.N., Hartley D.S. (2019). Distributed biomass supply chain cost optimization to evaluate multiple feedstocks for a biorefinery. Appl. Energy.

[73] Afedzi A.E.K., Rattanaporn K., Parakulsuksatid P. (2022). Impeller selection for mixing high-solids lignocellulosic biomass in stirred tank bioreactor for ethanol production. Bioresour. Technol. Rep..

[74] Kim G.Y., Yang J., Han Y.H., Seo S.W. (2024). Synthetic redesign of *Escherichia coli* W for faster metabolism of sugarcane molasses. Microb. Cell Fact..

[75] Stojkov V., Rakita S., Banjac V., Rakić D., Fišteš A., Vidosavljević S., Pinotti L. (2025). Optimising soybean molasses in broiler feed diet: Influence on pelleting process and pellet physical quality. Ital. J. Anim. Sci..

[76] Rodrigues B.C.G., de Mello B.S., Gonsales da Costa Araujo M.L., Ribeiro da Silva G.H., Sarti A. (2021). Soybean molasses as feedstock for sustainable generation of biomethane using high-rate anaerobic reactor. J. Environ. Chem. Eng..

[77] Zhang S., Wang J., Jiang H. (2021). Microbial production of value-added bioproducts and enzymes from molasses, a by-product of sugar industry. Food Chem..

[78] Rangaswamy V., Ramakrishna S.V. (2008). Lactic acid production by *Lactobacillus delbrueckii* in a dual reactor system using packed bed biofilm reactor. Lett. Appl. Microbiol..

[79] Calabia B.P., Tokiwa Y. (2007). Production of D-lactic acid from sugarcane molasses, sugarcane juice and sugar beet juice by *Lactobacillus delbrueckii*. Biotechnol. Lett..

[80] Guo S., Luo J., Wu Y., Qi B., Chen X., Wan Y. (2018). Decoloration of sugarcane molasses by tight ultrafiltration: Filtration behavior and fouling control. Sep. Purif. Technol..

[81] Zhang Z., Li D., Zhang X. (2019). Enzymatic decolorization of melanoidins from molasses wastewater by immobilized keratinase. Bioresour. Technol..

[82] Salatein N.M., Hassan R.K., Ibrahim R.A., Desouky S.E., El-Belely E.F., Abdel-Rahman M.A., Fahim I. (2025). Sustainable lactic acid production: Optimization yield from sugarcane and beet molasses through fermentation. Results Eng..

[83] Tang M., Yuan R., Huang G., Long Z., Liang X., Meng H., Long X. (2025). Toxicity mechanisms of metal ions in sugarcane molasses to *Saccharomyces cerevisiae*: Integrated physiological and transcriptomic insights. Food Biosci..

[84] Melikoglu M. (2026). Recent advancements in lactic acid production from food and other biowastes: A global review of biorefinery strategies and circular economy. Food Humanit..

[85] Vanapalli K.R., Bhar R., Maity S.K., Dubey B.K., Kumar S., Kumar V. (2023). Life cycle assessment of fermentative production of lactic acid from bread waste based on process modelling using pinch technology. Sci. Total Environ..

[86] Delmoitié B., Sakarika M., Rabaey K., De Wever H., Regueira A. (2025). Tailoring non-axenic lactic acid fermentation from cheese whey permeate targeting a flexible lactic acid platform. J. Environ. Manag..

[87] Cheng Q., Tao J., Li Y., Li W., Li D., Liu Y., Shi X., Liu X., Zhang X., Tong Y. (2021). Production of nisin and lactic acid from the starch of sweet potato by simultaneous saccharification and fermentation with two stage pH adjustment. 3 Biotech..

[88] Reddy G., Altaf M., Naveena B.J., Venkateshwar M., Kumar E.V. (2008). Amylolytic bacterial lactic acid fermentation—A review. Biotechnol. Adv..

[89] Shinkawa S., Okano K., Tanaka T., Ogino C., Kondo A. (2009). Efficient D-lactic acid production from raw starch. J. Biosci. Bioeng..

[90] Abdel-Rahman M.A., Sonomoto K. (2016). Opportunities to overcome the current limitations and challenges for efficient microbial production of optically pure lactic acid. J. Biotechnol..

[91] Panesar P.S., Kennedy J.F., Gandhi D.N., Bunko K. (2007). Bioutilisation of whey for lactic acid production. Food Chem..

[92] Mukherjee P., Sivaprakasam S. (2025). Metabolic engineering of *Lactobacillus delbrueckii* subsp. *bulgaricus* VI104 as a D-lactic acid cell factory through strategic pathway optimization for enhanced biosynthesis. World J. Microbiol. Biotechnol..

[93] Lips D. (2021). Fuelling the future of sustainable sugar fermentation across generations. Eng. Biol..

[94] Garofalo C., Norici A., Mollo L., Osimani A., Aquilanti L. (2022). Fermentation of microalgal biomass for innovative food production. Microorganisms.

[95] Tong K.T.X., Tan I.S., Foo H.C.Y., Lam M.K., Lim S., Lee K.T. (2022). Advancement of biorefinery-derived platform chemicals from macroalgae: A perspective for bioethanol and lactic acid. Biomass Convers. Biorefinery.

[96] Vázquez-Romero B., Perales J.A., Pereira H., Barbosa M., Ruiz J. (2022). Techno-economic assessment of microalgae production, harvesting and drying for food, feed, cosmetics, and agriculture. Sci. Total Environ..

[97] Zhang Y., Xu Z., Lu M., Ma X., Chen S., Wang Y., Shen W., Li P., Jin M. (2023). High titer (>200 g/L) lactic acid production from undetoxified pretreated corn stover. Bioresour. Technol..

[98] Wang H., Zhu B. (2024). Directed preparation of algal oligosaccharides with specific structures by algal polysaccharide degrading enzymes. Int. J. Biol. Macromol..

[99] Gong Y., Shang D.-D., Sun C.-L., Du Z.-J., Chen G.-J. (2024). Direct degradation of fresh and dried macroalgae by *Agarivorans albus* B2Z047. Mar. Drugs.

[100] Tůma S., Izaguirre J.K., Bondar M., Marques M.M., Fernandes P., da Fonseca M.M.R., Cesário M.T. (2020). Upgrading end-of-line residues of the red seaweed *Gelidium sesquipedale* to polyhydroxyalkanoates using *Halomonas boliviensis*. Biotechnol. Rep..

[101] Gu X., Zhou Y. (2026). Unlocking the potential of macroalgae: Innovative pretreatment strategies for efficient biorefinery. Molecules.

[102] Kaur A. (2025). Macroalgae-based biofuel production: Advances in cultivation, conversion pathways and techno-economic perspectives. Next Res..

[103] Sasaki Y., Yoshikuni Y. (2022). Metabolic engineering for valorization of macroalgae biomass. Metab. Eng..

[104] Chilakamarry C.R., Sakinah A.M.M., Zularisam A.W., Pandey A. (2021). Glycerol waste to value added products and its potential applications. Syst. Microbiol. Biomanuf..

[105] Qiu Y., Lei P., Zhang Y., Sha Y., Zhan Y., Xu Z., Li S., Xu H., Ouyang P. (2018). Recent advances in bio-based multi-products of agricultural *Jerusalem artichoke* resources. Biotechnol. Biofuels.

[106] Li Y., Wang L., Ju J., Yu B., Ma Y. (2013). Efficient production of polymer-grade D-lactate by *Sporolactobacillus laevolacticus* DSM442 with agricultural waste cottonseed as the sole nitrogen source. Bioresour. Technol..

[107] Okano K., Sato Y., Hama S., Tanaka T., Noda H., Kondo A., Honda K. (2022). L-lactate oxidase-mediated removal of L-lactic acid derived from fermentation medium for the production of optically pure D-lactic acid. Biotechnol. J..

[108] Beitel S.M., Sass D.C., Coelho L.F., Contiero J. (2016). High D(−) lactic acid levels production by *Sporolactobacillus nakayamae* and an efficient purification. Ann. Microbiol..

[109] Assavasirijinda N., Ge D., Yu B., Xue Y., Ma Y. (2016). Efficient fermentative production ofpolymer-grade D-lactate by an engineeredalkaliphilic *Bacillus* sp. strain under non-sterileconditions. Microb. Cell Fact..

[110] Wang L., Zhao B., Li F., Xu K., Ma C., Tao F., Li Q., Xu P. (2011). Highly efficient production of D-lactate by *Sporolactobacillus* sp. CASD with simultaneous enzymatic hydrolysis of peanut meal. Appl. Microbiol. Biotechnol..

[111] Świątkiewicz S., Arczewska-Włosek A., Józefiak D. (2016). The use of cottonseed meal as a protein source for poultry: An updated review. World’s Poult. Sci. J..

[112] Lv L., Xiong F., Pei S., He S., Li B., Wu L., Cao Z., Li S., Yang H. (2025). Synergistic fermentation of cottonseed meal using *Lactobacillus mucosae* LLK-XR1 and acid protease: Sustainable production of cottonseed peptides and depletion of free gossypol. Food Chem..

[113] Cruz-Casas D.E., Aguilar C.N., Ascacio-Valdés J.A., Rodríguez-Herrera R., Chávez-González M.L., Flores-Gallegos A.C. (2021). Enzymatic hydrolysis and microbial fermentation: The most favorable biotechnological methods for the release of bioactive peptides. Food Chem. Mol. Sci..

[114] Zhang Z., Yang D., Liu L., Chang Z., Peng N. (2022). Effective gossypol removal from cottonseed meal through optimized solid-state fermentation by *Bacillus coagulans*. Microb. Cell Fact..

[115] Wang S., Zhu L., Yu Z., Liang Q., Li D., Mou H. (2025). Detoxification and nutritional improvement of defatted cottonseed meal through sequential co-fermentation and the production of a low toxic cottonseed peptides. Ind. Crops Prod..

[116] Singh P., Krishnaswamy K. (2023). Non-GMO-high oleic soybean meal value addition and studying the functional and reconstitution behavior. Int. J. Food Prop..

[117] Zhang X., Du H., Xu Z., Wang Y., Guo X., Xiao H., Li Y. (2023). A novel alcalase-hydrolyzed soybean meal hydrolysates prepared using by-product material: Structure, function property, sensory property, and biological activity. Food Biosci..

[118] Suprayogi W.P.S., Ratriyanto A., Akhirini N., Hadi R.F., Setyono W., Irawan A. (2022). Changes in nutritional and antinutritional aspects of soybean meals by mechanical and solid-state fermentation treatments with *Bacillus subtilis* and *Aspergillus oryzae*. Bioresour. Technol. Rep..

